# Esophageal cancer surgery in Ethiopia: postoperative mortality and long-term survival

**DOI:** 10.1016/j.sipas.2025.100288

**Published:** 2025-06-03

**Authors:** Mintesinot Birhanu Senbeta, Sileshi Abiy, Hirbo Samuel, Nigist Birhanu

**Affiliations:** a Department of Anesthesiology, College of Medicine and Health Sciences, Dilla University, Dilla, Ethiopia; b Department of Anesthesia, College of Health Sciences, Addis Ababa University, Addis Ababa, Ethiopia; c Department of Pharmacy, College of Medicine and Health Sciences, Dilla University, Dilla, Ethiopia

**Keywords:** Esophageal cancer, Esophageal surgery, Ethiopia, Survival status

## Abstract

**Introduction:**

Cancer remains the leading cause of death worldwide, with esophageal cancer being the sixth leading cause of cancer-related deaths. For individuals with esophageal cancer, esophagectomy is the most effective treatment option available and has a high risk of both death and morbidity. Moreover, despite advances in preoperative optimization, surgery, and anesthesia techniques as well as the introduction of neoadjuvant therapy, the mortality and morbidity associated with esophagectomy remain high.

**Objective:**

To assess the survival status and predictors of postoperative survival in patients who underwent esophageal surgery for esophageal malignancies at selected hospitals from to 2018–2023 in Ethiopia, Addis Ababa.

**Methods:**

This was a retrospective cohort study. After acquiring data from the chart review, the data were analyzed using R version 4.3.3. Descriptive statistics for categorical variables were reported as frequencies and percentages. Kaplan-Meier curves and log-rank tests were used to estimate the survival curve and the difference in survival among groups within each covariate. After esophageal surgery, the impact of each covariate on time to death was assessed using the Cox proportional hazard model.

**Results:**

One hundred eighty-three patients who underwent esophageal surgeries for esophageal malignancy at four governmental hospitals in Addis Ababa over 5 years were included. The mean age was 53.8 years. 120 (65.6 %) had a tumor size <3.3 cm, and squamous cell carcinoma accounted for 154 (84.2 %) cases. Epidural analgesia was the most commonly used analgesic technique, 40 (21.9 %). The 30-day mortality was 10.9 %. The overall 1-, 2-, 3-, 4-, and 5-year survival rates were 53 %, 30.6 %, 19.5 %, 19.5 %, and 13 %, respectively. The median survival was 17 months. ASA score of > III (AHR = 2.14, 95 % CI: 1.12–4.12), cervical anastomotic leak (AHR = 3.29, 95 % CI: 1.44–7.52), and sepsis (AHR = 3.70, 95 % CI: 1.46–9.38) were identified as predictors of postoperative mortality in the multivariate Cox regression model.

**Conclusion:**

In Ethiopia, patients who underwent surgery for esophageal cancer had low 5-year survival rates.

## Introduction

Esophageal cancer is the sixth leading cause of cancer-related deaths and the seventh most commonly diagnosed cancer worldwide [1]. Despite recent breakthroughs in other therapeutic modalities, surgical excision remains the most effective treatment for patients with esophageal cancer and is often performed in conjunction with neoadjuvant chemotherapy or chemoradiotherapy [2].

At the Tikur Anbessa Specialized Hospital (TASH) in Addis Ababa, Ethiopia, esophageal cancer ranks ninth among patients with cancer [3]. and the 8th cause of cancer-related death among males [4]. Estimates of Cancer Incidence in Ethiopia in 2015 using population-based registry data showed that esophageal cancer was ranked 11th across five regions in Ethiopia in both males and females [5].

In Ethiopia, surgery is an independent predictor of survival among patients with esophageal cancer. Patients who underwent any form of esophageal surgery had a 30 % lower mortality rate compared to those who did not [6]. However, the survival outcomes of patients who have undergone esophageal surgery, as well as the predictors of postoperative mortality, remain unstudied.

This study aims to evaluate the impact of esophageal surgery on patient survival and investigate the influence of preoperative comorbidities and functional capacity, intraoperative anesthesia techniques and surgical events, postoperative pain management, and postoperative complications on postoperative mortality following esophageal resection.

## Methods and materials

### Study setting and period

For this study, a purposive sampling technique was employed to select four governmental hospitals: Tikur Anbessa Specialized Hospital, Menelik II Hospital, Saint Paul Millennium Medical College, and Saint Peter Specialized Hospital.

The medical records of esophageal cancer patients who underwent esophageal surgery between January 1, 2018, and December 30, 2022, at the four designated government hospitals were reviewed for data collection between March 27 and April 20, 2024. The follow-up period began at the time of esophageal surgery, with endpoints defined as December 30, 2023—the final day of follow-up—or the date of death, loss to follow-up, or last recorded contact.

### Study design and participants

A Retrospective Cohort Study was Conducted through a review of medical charts of patients who underwent esophagectomy for esophageal malignancies at four governmental hospitals.

All patients who underwent esophageal surgery for esophageal malignancy at the four selected governmental hospitals between 2018 and 2022 and met the eligibility criteria were included in this study. Patients with incomplete demographic or clinical information—such as age, sex, date of diagnosis, or type of treatment—were excluded.

A total of 408 patients were diagnosed with esophageal cancer and underwent esophageal surgery at the selected hospitals during the study period. Of these, 25 were excluded due to incomplete medical records, 63 were excluded because they received only feeding tube placement, and 5 patients had a different diagnosis. Additionally, 132 medical records could not be retrieved.

Consequently, a total of 183 patients were included in the final analysis.

### Data collection procedures

First, the total number of patients who underwent esophageal resection between January 1, 2018, and December 30, 2022, was identified using the operation theatre logbooks at each of the four selected hospitals. Subsequently, the corresponding medical records were retrieved, and relevant information was extracted.

Each selected medical chart was followed for a minimum of 30 days and a maximum of six years post-esophagectomy. Data were collected by two trained anesthesiology clinicians through a thorough review of patient charts and the cardiothoracic surgery registry, using a pretested and structured data extraction form (checklist).

The final outcome status of patients was determined by reviewing the medical records for a death certificate or death summary. In cases where this documentation was not available, follow-up phone calls were made to the patients or their attendants to ascertain survival status. During these interviews, additional data not available in the charts or registry were also collected.

Informed consent was obtained from all participants and/or their legal guardians.

### Data processing and analysis

After the data were acquired by reviewing the medical records of patients who underwent esophageal surgery using the Google Forms online platform, the data were exported and coded into numerical variables and analyzed using R Version 4.3.3 and RStudio Build 735. Categorical variables were summarized using frequency distribution and continuous variables were expressed in terms of dispersion and mean.

The dependent variable was dichotomized as death and censored. The Kaplan-Meier technique was used to create a survival table that revealed the likelihood of survival following successful surgery at different time points. In addition, a Kaplan-Meier Curve was generated to visually assess the survival probabilities, and the log-rank test was used to test the null hypothesis of no difference in survival between the two groups within each covariate. Cox regression analysis was performed to determine predictors of survival time. Variables (predictors) that fulfilled the assumption of proportional hazards and had a significant impact on time to death (i.e., Schonfeld test) were considered for bivariate Cox regression.

Bivariate Cox regression was carried out using each predictor variable and the time-to-death variable, and these independent variables that turned out to have an impact on time-to-death on the bivariable Cox regression at a p-value of less than or equal to 0.25 were included in the multivariable analysis. Multivariable Cox regression was performed at a 0.05 level of significance to determine the net effect of each predictor variable on time to death. The overall model fitness was checked using the C-Index, and the likelihood ratio test, Wald test, and log-rank test were used to assess the model fitness. The outcomes of these models are presented as hazard ratios (HRs) with a 95 % confidence interval, and statistically significant factors were found using P-values.

### Ethical approval

The research was conducted after obtaining ethical clearance from Addis Ababa University College of Health Sciences, Department of Anesthesia, Institutional Review Board with a grant number Anes/10/2023/2024. Informed consent was obtained from all participants and/or their legal guardians. The Information Acquired was used only for study purposes, and the privacy of each patient’s information was kept confidential. All procedures were performed in accordance with the relevant guidelines and regulations and met the guidelines listed in the WMA Declaration of Helsinki – Ethical Principles for Medical Research Involving Human Subjects.

## Results

### Sociodemographic and behavioral characteristics of study participants

A total of 183 patients underwent esophageal surgery, of whom 125 (63.8 %) were from rural areas. The mean age of the patients was 53.8 years (SD: 10.62), and 117 (63.9 %) were female. Eight patients (4.4 %) had a history of alcohol consumption, while five (2.7 %) reported a history of cigarette smoking.

Table 1, Table 2, Table 3, Table 4.

**Table 1:** Sociodemographic and behavioral characteristics of patients who underwent esophageal surgeries for esophageal malignancy at the 4 selected governmental hospitals, Addis Ababa, Ethiopia, 2018–2022.

Variables	Frequency	Percentage
Age (in Years) Mean-SD	53.8	10.62
Sex		
**Male**	66	36.1
**Female**	117	63.9
Residency		
**Urban**	58	31.7
**Rural**	125	68.3
History of Alcohol Intake		
**Yes**	8	4.4
**No**	175	95.6
History of Cigarette Smoking		
**Yes**	5	2.7
**No**	178	97.3

**Table 2:** Clinical stage, length of tumor, histologic types, anatomic location of esophageal cancer and types of treatments provided in patients who underwent esophageal surgeries for esophageal malignancy Addis Ababa, Ethiopia.

Variables	Frequency	Percentage
Site of Cancer		
**Upper Third**	1	0.5
**Middle Third**	32	17.5
**Lower Third**	115	62.8
**Gastroesophageal Junction**	35	19.2
Histology of Esophageal Cancer		
**Adenocarcinoma**	29	15.8
**Squamous Cell Carcinoma**	154	84.2
Size of Tumor		
**<3.3cm**	120	65.6
**>3.3cm**	63	34.4
Surgical Approach		
**Ivor Lewis**	35	19.1
**Trans Hiatal (Orringer)**	85	46.4
**Left Thoracoabdominal (Sweet)**	41	22.4
**Three Hole (McKeown)**	22	12.0
Induction Therapy		
**None**	181	98.9
**Chemo/Radiotherapy**	2	1.1

**Table 3:** Postoperative complications of patients who underwent esophageal surgeries for esophageal malignancy at the 4 selected governmental hospitals, Addis Ababa, Ethiopia, 2018–2022.

Variables	Frequency	Percentage
CARDIOVASCULAR		
Inotropic Support		
**Yes**	15	8.2
**No**	168	91.8
Cardiac Arrest		
**Yes**	6	3.3
**No**	177	96.7
PULMONARY		
Hospital Acquired Pneumonia		
**Yes**	26	14.2
**No**	157	85.8
Pneumothorax		
**Yes**	10	5.5
**No**	173	94.5
SURGICAL		
Cervical Anastomotic Leak		
**Yes**	19	10.4
**No**	164	89.6
Chylothorax		
**Yes**	4	2.2
**No**	179	97.8
OTHER COMPLICATIONS		
Septic Shock		
**Yes**	20	10.9
**No**	163	89.1
Renal Failure		
**Yes**	9	4.9
**No**	174	95.1
Reoperation		
**Yes**	21	11.5
**No**	162	88.5
ICU Admission		
**Yes**	53	29
**No**	130	71
MORTALITY		
Death within 30 Days		
**Yes**	20	10.9
**No**	163	89.1

**Table 4:** Predictors of operative survival in patients who underwent esophageal surgeries for esophageal malignancies.

Predictors	CHR (95 %CI)	AHR (95 %CI)
ASA PS Classification		
**>III**	2.38(1.36–4.16)⁎⁎	2.14(1.12–4.12)*
**<III**	Reference	
Postop Sepsis		
**Yes**	8.33(4.38–15.84)⁎⁎⁎	3.70(1.46–9.38)⁎⁎
**No**	Reference	
Postop Cervical Anastomotic Leak		
**Yes**	3.75(1.91–7.34)⁎⁎⁎	3.29(1.44–7.52)⁎⁎
**No**	Reference	

⁎ *P* < 0.05;.

⁎⁎ *P* < 0.01,.

⁎⁎⁎ *P* < 0.001.

### Physical and functional status of patients before surgery

Twenty three patients (12.6 %) had an ASA physical status classification score of ≥ 3, and 180 (98.4 %) had a functional capacity of ≥ 4 metabolic equivalents.

### Preoperative comorbidities

Cardiovascular diseases constituted the most prevalent comorbidities in the preoperative period. Hypertension was observed in 18 patients (9.8 %), and 9 patients (4.9 %) were receiving calcium channel blockers. Electrocardiographic abnormalities were detected in 46 patients (25.1 %), while 39 (21.3 %), 2 (1.1 %), 4 (2.2 %), and 6 (3.3 %) patients were diagnosed with Grade I diastolic dysfunction, pulmonary hypertension, pericardial effusion, and valvular heart disease, respectively.

Diabetes mellitus was identified in 3 patients (1.6 %), and HIV/AIDS was present in 7 patients (3.8 %). Additionally, 8 patients (4.4 %) reported preoperative cough symptoms, and 24 patients (13.2 %) exhibited decreased serum potassium levels.

### Preoperative nutritional status

A significant proportion of patients presented with Grade IV dysphagia. During preoperative evaluation, 88 patients (48.1 %) and 17 patients (9.3 %) had experienced a weight loss exceeding 10 % of their body weight in the preceding six months. Additionally, 56 patients (30.6 %) had hypoalbuminemia, while 20 patients (10.9 %) had a history of vomiting.

### Tumor characteristics of esophageal cancer and treatments provided for the patients

One hundred fifteen patients (62.8 %) had tumors located in the lower third of the esophagus. The majority of patients (120; 65.6 %) had a tumor size of <3.3 cm, and squamous cell carcinoma was the most common histological type, accounting for 154 cases (84.2 %).

The transhiatal (Orringer) approach was the most commonly performed surgical technique, used in 85 patients (46.4 %). Only 2 patients (1.1 %) received neoadjuvant therapy prior to surgery.

The majority of patients who underwent resection had a feeding tube placed during their hospital stay, which was subsequently removed prior to discharge.

### Postoperative complications

Complications classified as “other complications” were the most common during the postoperative period. These included ICU admission in 53 patients (29 %), septic shock in 20 (10.9 %), reoperation in 21 (11.5 %), renal failure in 9 (4.9 %), and tracheostomy in 2 (1.1 %).

Pulmonary complications included hospital-acquired pneumonia in 26 patients (14.2 %), pneumothorax in 10 (5.5 %), pleural effusion in 9 (4.9 %), and aspiration pneumonia in 3 (1.6 %).Surgical complications comprised cervical anastomotic leak in 19 patients (10.4 %), chylothorax in 4 (2.2 %), and wound dehiscence in 2 (1.1 %). Additionally, 11 patients (6 %) experienced intraoperative blood loss of ≥ 1000 ml. The 30-day mortality rate was 10.9 %. Notably, none of the patients developed an intrathoracic anastomotic leak.

### Survival rate of esophageal cancer patients who underwent esophageal surgeries

The 1,2-,3-,4- and 5-year Overall survival rates were 53 %, 30.6 %, 19.5 %, 19.5 %, and 13.0 %, respectively. The median survival duration was 17 months. A total of 74 patients died during the 1600 person-month follow-up period, resulting in an incidence rate of 46.25 per 1000 person-months.

**Fig. 1:**
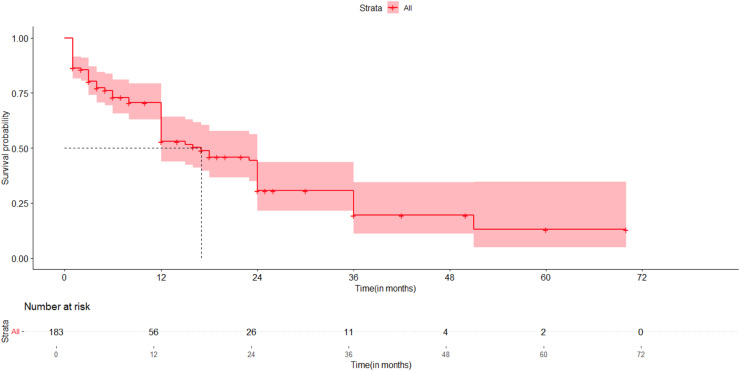
Kaplan-Meier curve of the overall survival pattern of patients who underwent esophageal surgeries for esophageal malignancy at the 4 selected governmental hospitals, Addis Ababa, Ethiopia, 2018–2022 GC.

**Fig. 2:**
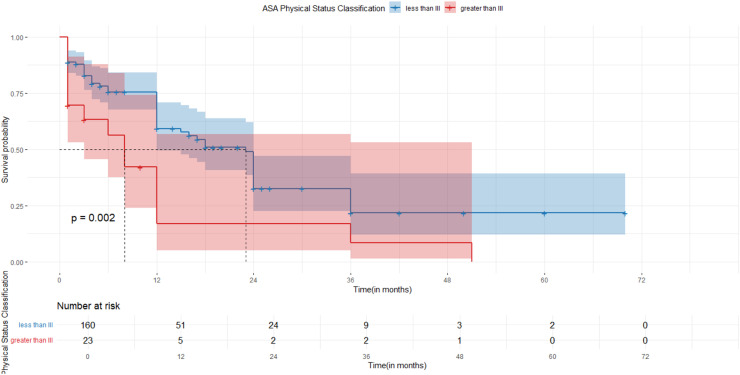
The difference in overall survival based on ASA PS Classification in patients who underwent esophageal surgeries for esophageal malignancy, Addis Ababa, Ethiopia (Log-rank test, *p* = 0.002).

**Fig. 3:**
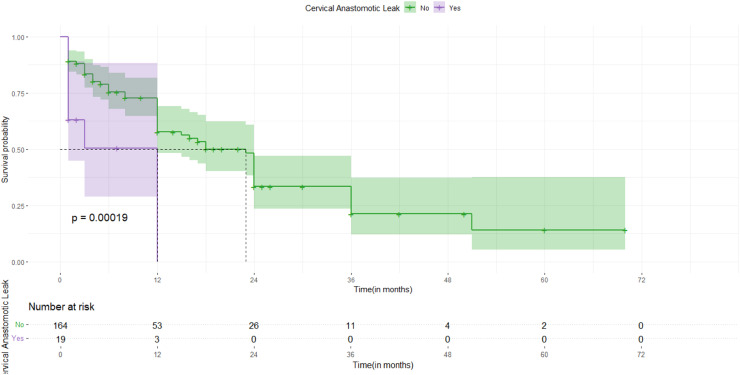
The Difference in overall survival based on cervical anastomotic leak in patients who underwent esophageal surgeries for esophageal malignancy, Addis Ababa, Ethiopia (Log-rank test, *p* < 0.00019).

Fig. 1, Fig. 2, Fig. 3.

### Variation in survival rates among groups

Individuals with an ASA Physical status score of <3 during the preoperative period had poor survival ((log-rank, *P* = 0.002)

Participants who developed cervical anastomotic leak had a lower survival rate than their counterparts (log-rank, *P* < 0.00019)

### Predictors of survival among esophageal cancer patients

Multivariate Cox proportional hazards regression identified several variables as significant predictors of survival following esophageal surgery. After adjusting for other covariates, patients with an ASA Physical Status (ASA PS) score of ≥ III in the preoperative period had a 2.14-fold increased hazard of death at any given time point compared to those with lower ASA scores (AHR = 2.14; 95 % CI: 1.12–4.12).

In addition, patients who developed cervical anastomotic leaks in the postoperative period were 3.29 times more likely to die compared to those without this complication (AHR = 3.29; 95 % CI: 1.44–7.52). Furthermore, the hazard of death increased by a factor of 3.70 among patients who developed systemic sepsis postoperatively, compared to their counterparts (AHR = 3.70; 95 % CI: 1.46–9.38).

## Discussion

In this multicenter cohort study, the overall survival status of patients who underwent esophageal surgery for malignancy at four selected hospitals was assessed, and the predictors of postoperative mortality were identified.

The 2-, 3-, and 5-year survival rates among these patients were found to be low, aligning with findings from a study conducted in South Africa, where the 5-year survival rate was reported as 13.2 % [7].

In contrast, the 3- and 5-year survival rates observed in this study are significantly lower compared to those reported in upper-middle-income countries. For instance, in Brazil, the 5-year survival rates are 56.6 % for squamous cell carcinoma and 58 % for adenocarcinoma [8], and China 3-year and 5-year survival rates of 43.7 % and 26.2 %, respectively [9]., the observed disparity may be explained by a combination of health system–related and disease-specific factors. In higher-income settings, earlier detection, timely referral, and greater access to multimodal treatment options—including neoadjuvant chemoradiotherapy, minimally invasive surgical techniques, and postoperative supportive care—contribute to improved outcomes.

Moreover, differences in infrastructure, such as the availability of diagnostic imaging, endoscopic facilities, oncology services, and specialized surgical expertise, play a critical role in enabling early-stage diagnosis and curative-intent interventions. In contrast, limited healthcare resources in low- and lower-middle-income settings may lead to delayed presentation, advanced disease at diagnosis, and restricted treatment options, all of which contribute to poorer long-term survival outcomes.

The overall survival rate was also lower than that in lower-middle income countries such as Iran 3 year and 5-year survival rates of 30.6 % and 21.3 %, respectively [10], and low-income countries such as Egypt, with a 3-year survival rate of 79.6 % [11] and Sudan’s 5-year survival rate of 21 % [12]. These variations in survival outcomes may be influenced by multiple factors, including genetic predisposition, dietary and nutritional patterns, cancer biology, and regional differences in public health infrastructure and healthcare-seeking behaviors. Additionally, disparities in the implementation of screening programs, access to early intervention, and treatment standardization may further contribute to the observed differences across countries with similar economic classifications.

In this study, the median survival time was 17 months in a study conducted in Maputo Hospital, South Africa it was 3.5 months. Conversely, the median survival time in this study was lower in Sudan, with a median survival time of 720 days (24 months).

The 30-day post-operative mortality rate in this study was 10.9 %. which is close to the mortality rate reported in Sudan (10 %) [12]. However, it is higher than the perioperative mortality rate of 6.5 % reported in a study conducted in Cairo. This discrepancy may be attributed to delayed hospital admission, with many patients presenting at advanced stages of disease, as well as the effects of long-standing nutritional deficiencies, both of which can compromise surgical outcomes and increase postoperative risk.

In this study, the likelihood of death increased by a factor of 2.14 at any given point in time in Patients with an ASA PS score of >III in the preoperative period compared to the other groups. (AHR = 2.14, 95 %CI 1.12–4.12). In two studies conducted in Japan, ASA-PS class was an independent prognostic factor for overall survival (HR= 3.259, 95 %CI = 1.588–6.685, *p* < 0.001). i.e. ASAIII compared to I and II.,) [13] and ASA physical status (P = 0.039, AHR 1.877 95 %CI 1.032–3.413) ASA 3 compared to ASA 1 or 2) [14]. Furthermore, research conducted in Germany is also consistent with our results ASA physical status IV (*P* < 0.0001, AHR=1.88,95 % CI (1.37–2.57) [15]. Since a higher ASA physical status classification shows a higher number of systemic diseases in a patient, creating an awareness of identification and early screening and treatment of non-communicable and communicable diseases as well as the deleterious effects of excessive drug use (alcohol, smoking, and other recreational drugs) habits on health is important, as this could potentially improve survival after esophageal surgery.

Furthermore, patients who developed cervical anastomotic leak in the postoperative period were 3.29 times as likely to die than those in the other groups. (AHR = 3.29, 95 %CI: 1.44–7.52). which is supported by studies conducted in Denmark(AHR = 2.37, 95 % CI (1.17–4.81), *P* = 0.016) [16] and Germany (*P* = 0.001, HR=2.29,95 % CI(1.43–3.69)) [15] The lower survival may be due to the probable immune suppression generated in the context of anastomotic insufficiency and subsequent local infection [17]. The resultant systemic inflammation may damage the cell-mediated immunity and notably the function of natural killer cells and cytotoxic T lymphocytes, encouraging the development and proliferation of micrometastases [18]. Hence, Timely initiation of postoperative chemotherapy is beneficial for decreasing tumor progression and improving survival; however, our results contradict the findings of a study conducted in Amsterdam [19] and Japan [13] where Anastomotic Leakage did not affect long-term survival. This difference may be explained by the advanced surgical techniques and access to early postoperative systemic management in these areas.

Finally, The Hazard of death was increased by a factor of 3.70 for patients who developed systemic sepsis in the postoperative period compared with their counterparts. (AHR = 3.70, 95 %CI: 1.46– 9.38). In a Danish study, sepsis/septic shock significantly decreased long-term survival (AHR=3.84, 95 %CI= (1.69–8.75), *p* < 0.001). This could be attributable to the individuals' heightened immunological vulnerability after surgery, as well as the activation of pathways, including IL-32 [20]. Hence, it is beneficial to cultivate the practice of the early initiation of empirical antibiotics to improve survival.

## Strength and limitation of the study

This study was conducted across four governmental hospitals in Addis Ababa, and the findings will provide a better picture of the survival status of patients in the country.

Participants were followed up for a long period, and a phone call was made to check for event status and information that could not be found in the medical records.

Finally, the inclusion of all patients who fulfilled the eligibility criteria allowed us to avoid or minimize sampling errors.

Selection bias is a potential problem, as patients with incomplete records were excluded during secondary data collection, which might have underestimated or overestimated the magnitude of death.

## Conclusion

In conclusion, this study found that the overall 5-year survival rate of patients who underwent esophageal surgery for malignancy was 13 %, with a median survival time of 17 months. The mean age of patients was 53.8 years, and the predominant histological type was squamous cell carcinoma. Esophageal cancer most commonly affected the lower third of the esophagus, and the transhiatal esophagectomy was the most frequently performed surgical approach.

In multivariate Cox regression analysis, an ASA Physical Status classification of ≥ III, cervical anastomotic leak, and septic shock were identified as significant predictors of increased postoperative mortality.

## Recommendation

The survival rate of patients who developed postoperative complications is low. Thus, thus screening, prevention, and early management of postoperative complications are recommended. In addition, health professionals from various disciplines should work together to devise and implement strategies to increase awareness of prevention, early detection, and timely and appropriate management of postoperative complications.

Patients with preoperative non-communicable disease had a lower survival rate; the same was true for patients with an ASA physical status of >3 Therefore, it is important to create public awareness of early symptoms and prevention strategies for these diseases to familiarize themselves with the habit of an early visit to a health care center.

**Table:** ABBREVIATION / ACRONYM.

AHR	Adjusted Hazard Ratio
ASA	American Society of Anesthesiologists
ASA PS	American Society of Anesthesiologists Physical Status
CHR	Crude Hazard Ratio
CI	Confidence Interval
IL-32	Interleukin-32
Post op	Postoperative
SD	Standard Deviation
TASH	Tikur Anbessa Specialized Hospital
WMA	World Medical Association

## Ethics approval and consent to participate

The research was conducted after obtaining ethical clearance from Addis Ababa University College of Health Sciences, Department of Anesthesia, Institutional Review Board with a grant number of Anes/10/2023/2024. Informed consent was obtained from all study participants. The Information Acquired was used only for study purposes, and the privacy of each patient’s information was kept confidential.

## Consent for publication

Not applicable.

## Availability of data and materials

The datasets used and/or analyzed during the current study are available from the corresponding author upon reasonable request.

## Funding

This research was financially supported by Addis Ababa University; however, the funders had no role in the study design, data collection and analysis, or manuscript preparation.

## CRediT authorship contribution statement

**Mintesinot Birhanu Senbeta:** Writing – review & editing, Writing – original draft, Visualization, Validation, Software, Resources, Methodology, Investigation, Funding acquisition, Formal analysis, Data curation, Conceptualization. **Sileshi Abiy:** Writing – review & editing, Writing – original draft, Validation, Supervision, Software, Methodology, Investigation, Formal analysis, Data curation, Conceptualization. **Hirbo Samuel:** Writing – review & editing, Writing – original draft, Validation, Supervision, Methodology, Investigation, Formal analysis, Data curation, Conceptualization. **Nigist Birhanu:** Writing – review & editing, Writing – original draft, Visualization, Validation, Supervision, Software, Methodology, Formal analysis, Data curation, Conceptualization.

## Declaration of competing interest

The corresponding author has received a financial support from Addis Ababa University to conduct this research. All authors declare that they have no known competing financial interests or personal relationships that could have appeared to influence the work reported in this paper.
